# Editorial: Seafood waste utilization: Isolation, characterization, functional and bio-active properties, and their application in food and nutrition

**DOI:** 10.3389/fnut.2022.948624

**Published:** 2022-07-14

**Authors:** Nilesh Prakash Nirmal, Sajid Maqsood

**Affiliations:** ^1^Institute of Nutrition, Mahidol University, Nakhon Pathom, Thailand; ^2^Department of Food Science, College of Agriculture and Veterinary Medicine, United Arab Emirates University, Al-Ain, United Arab Emirates

**Keywords:** seafood, waste utilization, bio-active compounds, functional properties, bio-activities

Seafood industries contribute to the global economy by supporting wellbeing as well as the nutritional status of the community. Generally, 30% of the total fish captured is unutilized owing to low-value discards, storage problems, and spoilage (1). The remaining harvested fish go through the processing plant where only 30–50% of fish are used as edible products and the remaining parts (70–50%) are discarded as by-products or leftovers (2). The by-products from the fish processing industry contain the skin, head, viscera, bones, scales, and fins, etc. These fish processing waste could be converted into marketable high-value products such as protein and peptides, oil and lipids, vitamins, minerals, pigments, and enzymes (2, 3). These active compounds exhibit various functional properties, biological activities, food fortification, and health benefits (2–4). In addition, waste utilization comes under one of the Sustainable Development Goals (SDGs) of the United Nations. Therefore, it is worth studying the chemical composition, extraction methods, purification and isolation, functional properties, biological activities, and industrial application (food, feed, nutraceutical, and pharma) of the bioactive compound obtained from seafood waste.

The goal of this Research Topic was to provide information about recent advancements in seafood waste utilization processes, the bioactive compounds present, and their various industrial and food applications. This provides more robust knowledge to researchers, food technologists and engineers, industry personnel, and academia for future research and the creation of a circular economy.

The Research Topic includes eight papers covering different aspects of seafood waste utilization. Seven of these articles report on the isolation and applications of different bioactive compounds from various fish and shellfish waste by-products. Furthermore, these bioactive compounds have been shown to exert numerous biological activities and nutraceutical benefits. Besides this, one of the studies focused on the development of nutritional crackers using jellyfish powder. Maisont et al. report that 30% replacement of wheat flour with jellyfish powder produces crackers with good physical and chemical characteristics as well as favorable scores in terms of whether people like them. Although jellyfish substitution enhances the nutritional composition of crackers, further studies are needed to optimize jellyfish concentration in order to minimize the negative effect on the crackers such as dark color and brittleness (Maisont et al.). In another study, shrimp oil was extracted from cephalothorax (CPO) and hepatopancrease (HPO) of Pacific white shrimp (*Litopenaeus vannamei*) and compared their chemical compositions, nutritional and quality indices (Raju et al.). Analyses report revealed that HPO with higher yield possesses a higher amount of astaxanthin, poly-unsaturated fatty acid, atherogenic index, and thrombogenicity index compared to the CPO. Hence HPO claimed to have more health benefits than CPO (Raju et al.).

The protein hydrolysate (PH) was prepared from skipjack tuna (*Katsuwonus pelamis*) roes using flavourzyme hydrolysis by Wang et al. PH was further subjected to ultrafiltration and column chromatography techniques to achieve 12 low molecular weight antioxidant peptides. The peptides were identified using Q-TOF-MS techniques as SGE, VDTR, AEM, QDHKA, TVM, QEAE, YEA, VEP, AEHNH, QEP, QAEP, and YVM (Wang et al.). Among the identified peptides, AEM, QDHKA, YEA, AEHNH, and YVM showed higher antioxidant activities and significantly protect against H_2_O_2−_ induced change in liver cells. In another study, Qiao et al. hydrolyzed tuna processing by-products (TPP) using neutrase enzyme. The hydrolysate was further purified and 14 anti-hypertensive peptides were identified using various chromatographic techniques. Among the 14 peptides, MWN, MEKS, MKKS, and LPRS possessed higher angiotensin-I-converting enzyme inhibitory activity as well as a protective effect on the human umbilical vein endothelial cells (HUVECs) by alleviating the negative effect of norepinephrine-induced nitric oxide production and endothelin-1 secretion (Qiao et al.). The above two studies indicate that bioactive peptides can be produced from tuna processing byproducts and could be used as natural sources of antioxidants.

An *in-silico* study has been performed to identify the potent bioactive compounds from seaweed waste for histone deacetylases-2 (HDAC-2) inhibition for cancer prevention (Bharadwaj et al.). Various sophisticated and advanced techniques have been utilized for the screening of 193 bioactive compounds from seaweed against the HDAC-2. The potent bioactive compound identified was cinnamyl dihydrocinnamate. This finding suggests that further exploration of seaweed-based bioactive compounds as HDAC-2 inhibitors for cancer treatment (Bharadwaj et al.) through *in-vitro* and *in-vivo* studies should be undertaken to confirm the drug-like properties of the identified compound against cancer.

This special issue includes three review articles on different aspects of seafood waste utilization. The first review article focuses on the valorization of aquaculture waste using biofloc technology and the aquamimicry concept (Nisar et al.). These novel approaches are largely used for tilapia and shrimp culture and can be used for semi-intensive and intensive aquaculture systems. The authors provide detailed information on biofloc formation, nutritional value, the effects on aquaculture microbiome, economic aspects, sustainability approaches, and application of biofloc technology. The newer concept of aquamimicry has also been described and compared with biofloc technology. Indeed, these two concepts provide an eco-friendly, sustainable approach to aquaculture production while minimizing land and water resources (Nisar et al.). The second mini-review provides information on the cold-adapted protease derived from fish waste and their application in fisheries and aquaculture waste utilization (Khiari). Herein, the author provided the different types of cold-adapted protease and their isolation process. Additionally, Khiari described the various industrial applications of cold-adapted proteases and their advantage in seafood waste utilization. The last review article provides a comprehensive discussion on the valorization of seafood discard to obtain various bioactive molecules including enzymes, collagen and gelatin, protein and peptides, lipid and carotenoids, polysaccharides, and their nutritional and nutraceutical benefits (Nag et al.). The authors also elaborated on the challenges and future perspectives in seafood waste utilization.

Seafood processing waste creates environmental contamination, economic burden, and health hazards as well as resulting in the loss of valuable compounds. However, this waste consists of a potential bioactive molecule that can be extracted and utilized for various industrial applications. The work published in the special issue provided a clear message that seafood waste is a rich source of various bioactive and nutritionally valuable compounds which have been utilized for novel food product development with various health benefits for food fortification and as nutritional and functional ingredients Nevertheless, new *in-silico* technologies can further help to identify the potent bioactive compound from the seafood discard. Additionally, with the help of new technology such as biofloc and aquamimicry, the land and water resource could be reduced while sustaining eco-friendly aquaculture production. Finally, seafood waste is a valuable resource with the potential to support the world economy and nutritional needs, which need more robust technologies and studies to confirm their benefits.

## Author Contributions

NN: wrote—concept, design, and draft the article. SM: writing, review and editing. All authors have read and agreed to the published version of the manuscript.

## Conflict of interest

The authors declare that the research was conducted in the absence of any commercial or financial relationships that could be construed as a potential conflict of interest.

## Publisher's Note

All claims expressed in this article are solely those of the authors and do not necessarily represent those of their affiliated organizations, or those of the publisher, the editors and the reviewers. Any product that may be evaluated in this article, or claim that may be made by its manufacturer, is not guaranteed or endorsed by the publisher.

## References

[1] FAO. The State of World Fisheries and Aquaculture 2020. Sustainability in Actions. Rome: FAO (2020).

[2] Nirmal NP Santivarangkna C Rajput MS Benjakul S Maqsood S. Valorization of fish byproducts: sources to end-product applications of bioactive protein hydrolysate. Compreh Rev Food Sci Food Saf . (2022) 21:1803–42. 10.1111/1541-4337.12917 35150206

[3] Nirmal NP Santivarangkna C Rajput MS Benjakul S. Trends in shrimp processing waste utilization: an industrial prospective. Trends Food Sci Technol. (2020)103:20–35. 10.1016/j.tifs.2020.07.001

[4] Nirmal NP Santivarangkna C Benjakul S Maqsood S. Fish protein hydrolysates as a health-promoting ingredient—recent update. Nutr Rev. (2021) 80:1013–26. 10.1093/nutrit/nuab065 34498087

